# Recent Trends in Photoacoustic Imaging Techniques for 2D Nanomaterial-Based Phototherapy

**DOI:** 10.3390/biomedicines9010080

**Published:** 2021-01-15

**Authors:** Woo Yeup Jeong, Moon Sung Kang, Haeni Lee, Jong Hun Lee, Jeesu Kim, Dong-Wook Han, Ki Su Kim

**Affiliations:** 1 School of Chemical Engineering, College of Engineering, Pusan National University, Busan 46241, Korea; duq5315@naver.com; 2 Department of Cogno-Mechatronics Engineering, College of Nanoscience & Nanotechnology, Pusan National University, Busan 46241, Korea; mskang7909@gmail.com (M.S.K.); dovelet2@pusan.ac.kr (H.L.); 3 Department of Food Science and Biotechnology, Gachon University, Seongnam, Gyeonggi 13120, Korea; foodguy@gachon.ac.kr

**Keywords:** 2D materials, phototherapy, photoacoustic imaging, image-guided therapy

## Abstract

A variety of 2D materials have been developed for therapeutic biomedical studies. Because of their excellent physicochemical properties, 2D materials can be used as carriers for delivering therapeutic agents into a lesion, leading to phototherapy. Various optical imaging techniques have been used for the monitoring of the treatment process. Among these, photoacoustic imaging has unique advantages including relatively deep imaging depth and large field of view with high spatial resolution. In this review article, we summarize the types of photoacoustic imaging systems used for phototherapy monitoring, then we explore contrast-enhanced photoacoustic images using 2D materials. Finally, photoacoustic image-guided phototherapies are discussed. We conclude that 2D material-based phototherapy can be efficiently monitored by photoacoustic imaging techniques.

## 1. Introduction

**Figure 1:**
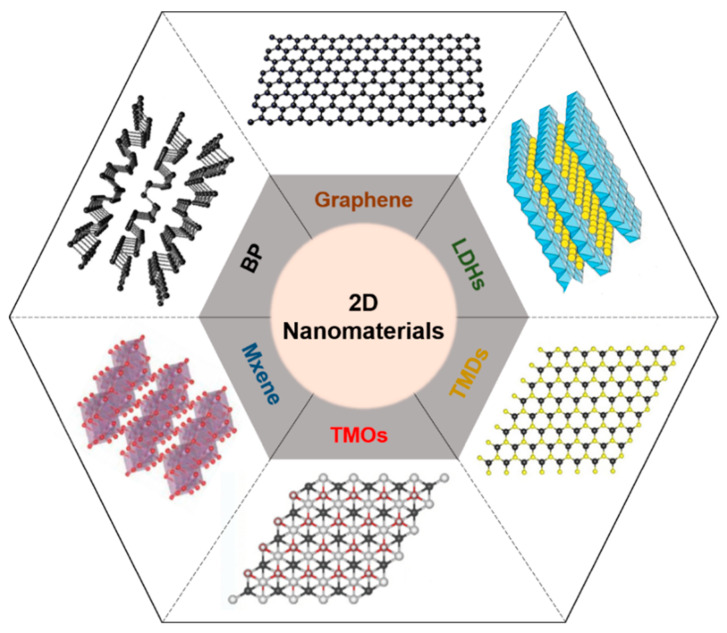
Schematic illustration of representative 2D nanomaterials. LDH, layered double hydroxide; TMD, transition metal dichalcogenide; TMO, transition metal oxide; BP, black phosphorus.

Two-dimensional nanomaterials with a well-ordered 2D planar structure (thickness > 100 nm) have been widely developed. Thanks to their beneficial biocompatible and biodegradable characteristics, various types of 2D nanomaterials, such as graphene derivatives; LDH, layered double hydroxide; TMD, transition metal dichalcogenide; TMO, transition metal oxide; and BP, black phosphorus, have been used for biomedical applications including drug delivery, tissue engineering, bio-imaging, and bio-sensing [1] (Figure 1). Because such materials have beneficial physicochemical properties of biocompatibility and degradability, they are suitable for biomedical applications including drug delivery, tissue engineering, bio-imaging, and biosensors [2,3,4,5,6]. Two-dimensional nanomaterials can cause a photothermal effect that generates heat by converting light energy into thermal energy when they are irradiated with near-infrared (NIR) light, and can then be used for phototherapy [7].

The representative phototherapy methods are photothermal therapy (PTT) and photodynamic therapy (PDT) [8]. In PTT, the local heating of NIR-absorbing agents is triggered by NIR light illumination. As tumor cells have difficulty dissipating heat, the NIR-triggered photothermal effect causes selective death of cancerous cells, which can be ablated more than 42 °C through necroptosis and apoptosis, which is programmed cell death, while endowing little damage to normal cells [9]. In contrast, PDT is an indirect method using photosensitizers that generate harmful singlet oxygen (^1^O_2_) when they absorb light. As PDT does not generate heat, nanomaterials in PDT usually perform as carriers that transfer photosensitizers using their surface properties [10]. To assess the therapy, drug delivery, and biodegradability, visualization of the internal biodistribution is essential.

Various biomedical imaging techniques, such as X-ray computed tomography (CT), magnetic resonance imaging (MRI), and positron emission tomography (PET), have been used for visualizing the distribution of nanomaterials, monitoring the delivery of nanomaterials, and assessing the efficacy of treatments [11,12,13,14]. Particularly, optical imaging techniques have been widely used as they provide high optical contrast, rich functional information, and excellent spatiotemporal resolution [15]. Compared with other biomedical imaging techniques, optical imaging systems can be implemented with low cost and simple configuration. In addition, optical imaging does not create harmful ionizing radiation, which makes the system favorable for future clinical translation. However, despite the advantages set out above, optical imaging is not widely used in clinics. The primary limitation of optical imaging is shallow imaging depth due to photon scattering in biological tissue [16].

**Figure 2:**
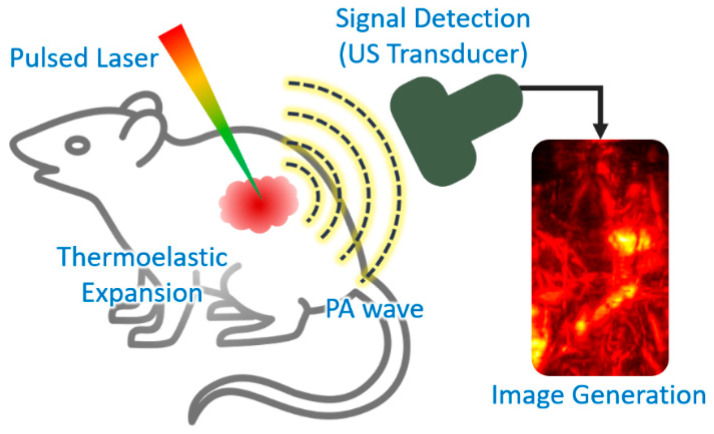
Schematic explanation of the procedure of PA imaging. PA, photoacoustic; US, ultrasound. The inset image is reproduced with permission from [27].

Photoacoustic (PA) imaging is a biomedical imaging technique that combines the principles of ultrasound (US) and optical imaging [17]. The principle of PA imaging is based on the PA effect, which involves energy transduction from laser to acoustic waves through thermoelastic expansion (Figure 2). The typical procedure of PA imaging is as follows: (1) illumination of a short (typically a few nanoseconds) pulsed laser beam to target tissue, (2) light absorption and heat release by the optically absorbing chromophores, (3) acoustic wave (i.e., PA wave) generation through rapid thermal expansion and relaxation, (4) signal reception using US transducer, and (5) image generation and display. Because PA imaging inherits the principles of optical and US imaging techniques, it can provide both strong optical contrast and high ultrasound resolution in deep tissue [18,19]. In addition to intrinsic chromophores (oxy- and de-oxy-hemoglobins, melanin, lipids, and water), external agents (organic dyes, liposomal nanoformulations, nanoparticles, and nanostructures) have been widely used to obtain contrast-enhanced PA images [20,21,22,23]. Moreover, using multiple wavelengths of the excitation laser, molecular functional information of biological tissue can be obtained, which can be used for investigating the bio-distribution of external agents in vivo [24,25,26].

In this review article, we summarize several types of preclinical PA imaging systems for monitoring the distribution of nanomaterials in vivo. We also introduce recent trends and therapeutic applications of PA imaging for 2D material-based phototherapy. By monitoring the delivery and theranostic procedure, we can determine the future direction of PA imaging as an investigational tool for 2D nanomaterials.

## 2. Photoacoustic Imaging System

In general, PA imaging systems can be classified as PA microscopy (PAM) and PA computed tomography (PACT). The main difference between the two types of system is mathematical computation in the image reconstruction algorithm. In PAM, a single-element transducer is used to achieve PA signals along the depth direction (i.e., A-line signal) at the top of the sample, thus no mathematical reconstruction algorithm is required. In contrast, PACT utilizes rotational scanning methods or multi-element US transducers to acquire tomographic information regarding the target. Therefore, PACT requires image reconstruction algorithms [28,29,30]. Compared with PACT, PAM systems have higher resolutions, but suffer from shallow imaging depth and slow imaging speed.

### 2.1. Photoacoustic Microscopy

**Figure 3:**
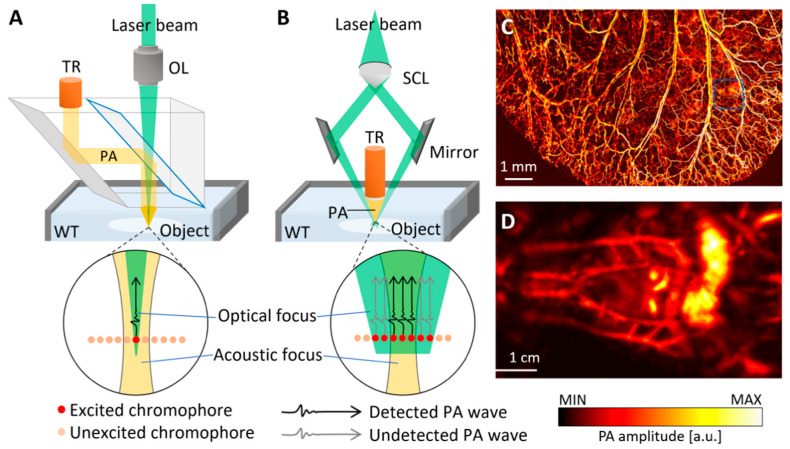
Schematic illustration and representative images of OR- and AR-PAM systems. The typical configurations of (**A**) OR-PAM and (**B**) AR-PAM. The optical focus is smaller than the acoustic focus in OR-PAM, while the optical focus is larger than the acoustic focus in AR-PAM. The red and orange dots are excited and unexcited chromophores, respectively. The black and gray arrows are detected and undetected PA waves, respectively. (**C**) PA images of mouse ear acquired using an OR-PAM. (**D**) PA images of the whole body of mouse acquired using an AR-PAM. OR-PAM, optical resolution photoacoustic microscope; AR-PAM, acoustic resolution photoacoustic microscope; PA, photoacoustic; TR, ultrasound transducer; WT, water tank; OL, objective lens; SCL, spherical conical lens. The images are reproduced with permission from [31,39].

PAM can be divided into two types: optical-resolution PAM (OR-PAM) and acoustic-resolution PAM (AR-PAM). OR-PAM uses tight optical focus, ~10 times smaller than acoustic focus, while the emphasis of AR-PAM is to tighten the acoustic focus. In OR-PAM, the lateral resolution is determined by the optical focal spot size, which depends on the numerical aperture of an objective lens (Figure 3A). The tightly focused optical beam excites optically absorbing chromophores only in the focal zone (red dot in Figure 3A), and the PA waves are generated from the excited chromophores. Because the optical focal spot is smaller than the acoustic focal spot, all the generated PA waves are detected by the US transducer (black arrow in Figure 3A). We can note that PA waves are not generated from the unexcited chromophores (orange dots in Figure 3A), which leads to the better resolution of OR-PAM compared with AR-PAM. In contrast, the resolution of AR-PAM is determined by the acoustic focal size because the optical focal spot is larger than the acoustic focal spot (Figure 3B). The size of the acoustic focal spot depends on the characteristics of the US transducer. The widely delivered optical beam excites chromophores in a large area (red dots in Figure 3B), then generates PA waves (black and gray arrows in Figure 3B). Among them, the PA waves in the acoustic focal zone (black arrows in Figure 3B) are detected by the ultrasound transducer. Therefore, the acoustic focus determines the resolution of the system.

By adjusting the optical focus, the resolution of OR-PAM can be greatly improved (Figure 3C) [31,32,33,34,35]. However, imaging depth can be limited because maintaining tight optical focus is difficult while scanning the imaging area. Therefore, compared with AR-PAM, OR-PAM systems usually have a small field of view (FOV) and a shallow imaging depth. As a tight optical focus is not required, AR-PAM has the advantage of being able to image deeper and larger areas in biological tissues compared with OR-PAM (Figure 3D) [36,37,38,39,40,41].

### 2.2. Photoacoustic Computed Tomography

**Figure 4:**
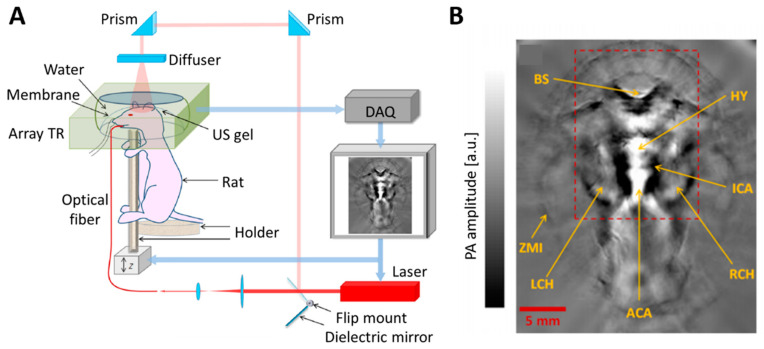
Schematic illustration and representative images of a PACT system. (**A**) Typical configurations of PACT. Array transducer is used to acquire PA images. (**B**) Representative PACT images of mouse brain in vivo. PA, photoacoustic; PACT, photoacoustic computed tomography; TR, ultrasound transducer; US, ultrasound; DAQ, data acquisition module; BS, brain stem; HY, hypothalamus; ICA, internal carotid artery; LCH, left cerebral hemisphere; RCH, right cerebral hemisphere; ACA, anterior cerebral artery; ZMI, zygomatic muscle interface. The images are reproduced with permission from [42].

For further enhancement of FOV and imaging speed, PA computed tomography (PACT) using array transducers has been introduced (Figure 4) [42,43,44,45]. Using multi-element transducers with various geometrical structures (linear, arc, planar, or hemispherical arrays), PACT can achieve PA signals at multiple positions with a single laser shot; therefore, the imaging speed and FOV can be significantly enhanced [45,46,47,48,49]. For potential clinical applications, clinical US machines have been used for PACT. As the receiving signals of both PA and US are acoustic waves, the signal acquisition and image reconstruction procedures of the two imaging techniques are identical, and they can thus share a single imaging platform [27,50,51,52]. Combined PA and US images, providing complementary structural and functional information regarding biological tissues, have been investigated in clinical applications such as melanoma detection, cancer classification, and vascular disease assessment [53,54,55,56].

### 2.3. Performance Benchmarks of Photoacoustic Imaging Systems

As each type of system has complementary advantages in terms of imaging depth, spatial resolution, imaging speed, and FOV, a variety of PA imaging systems have been applied in biomedical studies including the monitoring of phototherapy. The typical performances of PA systems are summarized in Table 1. OR-PAM has an excellent spatial resolution in a shallow depth of biological tissue, thus it is usually utilized for visualization of superficial regions such as the ear, eye, or peripheral vasculature. AR-PAM can obtain a signal that penetrates deeper into biological tissue and an extended FOV compared with OR-PAM, as is used for monitoring the whole-body distribution of nanomaterials, drugs, or contrast agents. PACT further extends FOV and imaging speed, thus it can monitor the real-time response of biological tissue during treatment using nanomaterials.

## 3. Phototherapy Using 2D Nanomaterials

### 3.1. Types and Characteristics

Currently, various phototherapy agents including gold nanostructures [57,58], mesoporous silica nanoparticles [59,60], and 2D nanomaterials have been widely investigated for clinical application. Among them, 2D nanomaterials are attracting the most attention because they can be used not only for phototherapy, but in other biomedical fields thanks to their excellent biochemical properties (Table 2). Typical 2D nanomaterials consist of one or a few atomic layers. As a representative 2D nanomaterial, graphene has a honeycomb structure consisting of carbon atoms. By changing the shape, the number of layers, and chemical modifications, several derivatives can be formed. Among them, graphene oxide (GO) [57] and reduced graphene oxide (rGO) [58,59] have been widely utilized for biomedical applications because of their superior electrical and thermal conductivities, large surface area, chemical versatility, and biocompatibility [60,61,62]. Layered double hydroxides (LDHs) are inorganic 2D nanomaterials with structures in which metal atoms are sandwiched by hydroxide layers. Because of their high charge density with excellent biocompatibility, LDHs are widely used as nano-carriers for theranostic agents in drug or gene delivery, phototherapy, and immunotherapy [63,64,65]. Transition metal dichalcogenides (TMDs) have hexagonal lattices consisting of monolayers of transition metal atoms between chalcogen atom layers. In addition to thin, flexible, and strong characteristics, TMDs are excellent optical absorbers, thus producing photolimunescence and photothermal effects [66,67,68,69]. TMDs can be formed with various materials including molybdenum disulfide (MoS_2_) [70,71,72,73], tungsten disulfide (WS_2_) [74,75,76,77], and molybdenum diselenide (MoSe_2_) [78,79,80]. Transition metal oxides (TMOs) are compounds of oxygen atoms and transition metals such as titanium (TiO_2_) [81,82] and manganese (MnO_2_) [83,84,85]. Their wide bandgap results in excellent photochemical and electric properties [86,87]. They can also directly interact with drugs, genes, or other biomolecules by surface modification and thus can be utilized in biomedical applications including drug delivery, cancer therapy, tissue engineering, bio-imaging, and biosensing [88,89,90]. Mxenes are the most recently discovered 2D materials and have been applied in various biomedical applications because of their extreme thinness, large surface area, high surface–volume ratio, and mechanical strength [91,92,93,94,95]. Black phosphoruses (BP) are the most stable allotropes of phosphorus with zigzag or armchair bilayer structures. BP have shown potential in biomedical applications with their strong optical absorption in the ultraviolet (UV) and NIR regions [96,97,98,99,100]. They have also demonstrated promising biocompatibility and biodegradation [101].

### 3.2. Phototherapy Using 2D Nanomaterials

**Figure 5:**
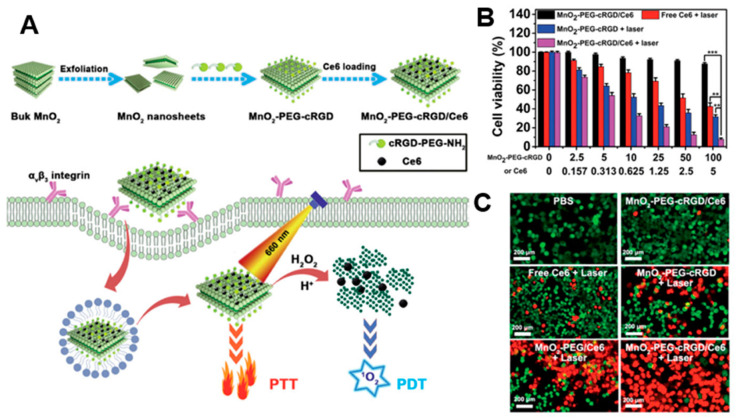
(**A**) Schematic illustration for the preparation of MnO_2_-PEG-cRGD/Ce6 for synergistic photothermal/photodynamic (PTT/PDT) therapy. (**B**) Relative viabilities of PC3 cells after incubation with free Ce6, MnO_2_-PEG-cRGD with 660 nm light irradiation, or MnO_2_-PEG-cRGD/Ce6 with or without 660 nm light irradiation (0.6 W cm^−2^, 10 min, *** *p* < 0.001, ** *p* < 0.01). (**C**) Fluorescence images of calcein acetoxymethyl ester (Calcein-AM, green)/propidium iodide (PI, red) double stained cells after different treatments. The images are reproduced with permission from [103].

By taking advantage of their strong optical absorption and thermal transition properties, 2D nanomaterials have been used for PTT. Liu et al. successfully prepared a doxorubicin (DOX)-loaded MoS_2_-PEG nanosheet for combined PTT–chemo cancer therapy [102]. In that study, nanosheets were analyzed for drug delivery and photothermal effects by NIR irradiation. MoS_2_-PEG/DOX nanosheets exhibited synergistic anti-cancer effects, inhibiting tumor growth in in vivo experiments. Zeng et al. reported on ultrathin MnO_2_ nanosheets of polyethylene glycol-cyclic arginine-glycineaspartic acid tripeptide (PEG-cRGD) and encapsulated chlorin e6 (Ce6) for PTT/PDT synergistic cancer therapy (Figure 5) [103]. In that study, the nanosheets showed photothermal efficiency of 39% and could be reduced by overexpressed acidic H_2_O_2_, which could efficiently generate O_2_ and further enhance the therapeutic efficiency of PDT. Moreover, the MnO_2_-PEG-cRGD/Ce6 exhibited pH-controlled and NIR-induced Ce6 release, and showed favorable therapeutic outcomes under a single 660 nm NIR laser.

**Figure 6:**
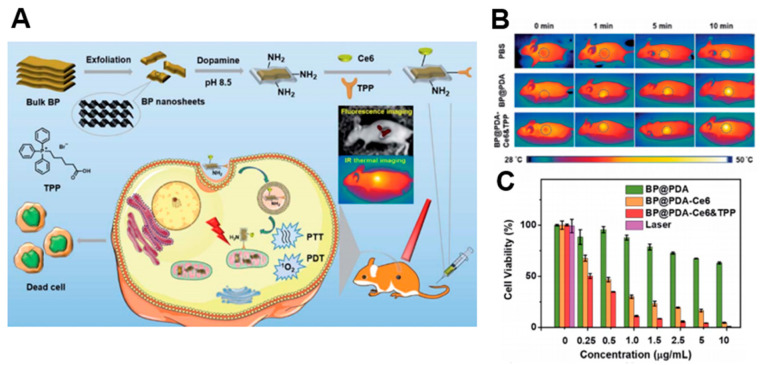
(**A**) Schematic illustrations of the preparation, therapeutic uses, and peaks in functions of BP@PDA–Ce6&TPP NSs. (**B**) Ex vivo fluorescence imaging of main organs as well as tumor at 24 h post-injection. (**C**) Relative viabilities of HeLa cells incubated with BP@PDA NSs, BP@PDA–Ce6 NSs, and BP@PDA–Ce6&TPP NSs at different BP@PDA concentrations with laser illumination (660 nm, 0.5 W cm^−2^, 5 min). Data represent mean ± SD (*n* = 4). The images are reproduced with permission from [104].

A number of PDT studies have investigated the development of 2D nanomaterials that release photosensitizers. Moosavi et al. prepared N-TiO_2_ nanoparticles to generate reactive oxygen species (ROS) and induce autophagy [81]. They showed the dose-dependent capability of well-dispersed photo-activated N-TiO_2_ NPs to induce terminal megakaryocyte differentiation and cell death in K562 leukemia cells. In cellular experiments, N-TiO_2_ nanoparticles increased ROS levels with light irradiation. Yang et al. fabricated covalently, incorporating both chlorin e6 (Ce6) and triphenyl phosphonium (TPP) onto BP@PDA NSs for dual-modal imaging-guided synergistic photothermal and photodynamic therapy (Figure 6) [104]. BP@PDA–Ce6&TPP NSs can produce considerable heat, mainly owing to BP@PDA NSs, and can generate sufficient ROS for PDT. With these results, BP@PDA–Ce6&TPP NSs can be used for PTT/PDT therapy of cancers.

**Table 2:** Phototherapy using 2D nanomaterials. PTT, photothermal therapy; PDT, photodynamic therapy.

2D Nanomaterials	Photothermal Conversion	Therapy	Applied Forms	Ref.
Graphene derivatives	63% (G), 35% (GO) [59]	PTT, PDT	GO-UCNPs-ZnPc	[61]
		PTT	GO/MnFe_2_O_4_/DOX	[62]
TiO_2_	40.8% [86]	PTT	Ag@TiO_2_	[82]
		PDT	N-TiO_2_	[81]
MoS_2_	0.84% [67]	PTT	MoS_2_-HA-DTPA-Gd	[71]
		PTT, PDT	AuNBPs@MoS_2_	[72]
		PTT	MoS_2_-Gd-BSA	[73]
BP	30.84% [97]	PTT	BP-Au NSs	[98]
		PTT	BP-PEG-FA/Cy7 NSs	[99]
		PTT, PDT	BP@PEG/Ce6 NSs	[100]
Mxene(Ti_3_C_2_)	≒100% [92]	PTT	Ti_3_C_2_@Au	[93]
		PTT, PDT	Ti_3_C_2_-SP	[94]
		PTT, PDT	Ti_3_C_2_-DOX	[95]
WS_2_	35% [68]	PTT, PDT	BSA-WS_2_@MB	[75]
		PTT	WS_2_-PEG	[76]
		PTT	WS_2_-IO/S@MO-PEG	[77]
MoSe_2_	54.3% [69]	PTT, PDT	MoSe_2_/Fe_3_O_4_	[79]
		PTT, PDT	MoSe_2_@PEG-Dox	[80]
2D Boron	42.5% [104]	PTT, PDT	B@Ce6–PAH–PAA	[105]
MnO_2_	62.4% [87]	PTT	MnO_2_-PEG-FA/DOX	[84]
		PTT	BSA-MnO_2_ NPs	[85]

## 4. Photoacoustic Image-Guided Phototherapy

### 4.1. Contrast-Enhanced Photoacoustic Imaging Using 2D Nanomaterials

PA imaging, one of the latest and most promising imaging modalities, has been shown to enable visualization of tissues with centimeter-depth penetration for in vivo imaging (Table 3). However, the optical contrast ratio of tissues is generally low; therefore, conventional PAI can only detect specific tissues that have strong absorbance. However, certain kinds of imaging probes can be utilized to enhance the optical contrast ratio of tissues with low absorbance. Specifically, incorporating 2D NMs that strongly absorb light in the tissues to be irradiated can overcome the inherently low contrast problem. When incorporating 2D NMs exhibiting PT properties into tissues with low contrast, they absorb light more than the surrounding tissue components, hence emitting a stronger PA signal.

**Figure 7:**
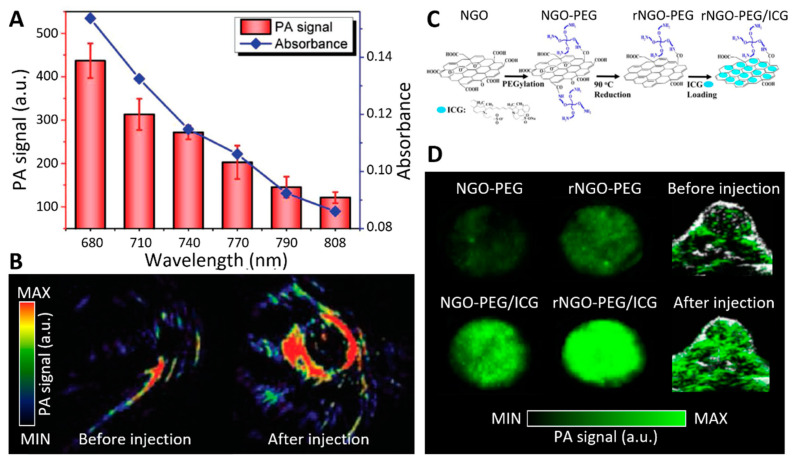
(**A**) In vitro PA signals and optical absorbances of TiL_4_-coordinated black phosphorus quantum dots (BPQDs) with various excitation wavelengths. (**B**) In vivo PA images of MCF-7 tumor xenografted Balb/c mice before and after intravenous injection of TiL_4_-coordinated BPQDs. (**C**) Schematic diagram of ICG-rGO nanocomposite preparation. (**D**) In vitro and in vivo PA signals of ICG-rGO nanocomposites with an excitation wavelength of 780 nm. The in vivo images of HeLa tumor inoculated Balb/c nude mice were acquired before and after tail vein injection of rNGO-PEG/ICG. PA, photoacoustic; NGO, nano-graphene oxide; rNGO, reduced nano-graphene oxide; PEG, polyethylene glycolated; ICG, indocyanine green. The images are reproduced with permission from [103,107].

BP is a newly emerging 2D nanomaterial that exhibits a layer-dependent bandgap, resulting in versatile properties for electronic and optoelectronic application. However, BP is highly reactive to oxygen and water, hence its physical properties, biodistribution, and optical properties can be altered in an in vivo environment. Several studies have focused on the stabilization of BP for PA applications. Sun et al. fabricated TiL4-coordinated BPQDs (TiL_4_@BPQDs) for PA imaging of cancer [103]. TiL_4_ enhanced the stability of BPQDs in aqueous dispersions and maintained optical properties for a prolonged time compared with bare BPQDs. A PA signal was observed from the MCF-7 cells after the addition of only 12.5 ppm of TiL4@BPQDs, which is only 0.63% of the common imaging dose of 2 mg/L, suggesting their high sensitivity in in vitro PA imaging (Figure 7A). Moreover, the in vivo PA images indicated a sixfold increase in PA signal at 680 nm after injection of TiL4@BPQDs into xenografted tumors in mice (Figure 7B), which was superior to the signal generated by Au nanorods (a common PA exogenous probe). These results suggest the potential of BP for PA imaging if the unstable in vivo properties can be resolved by surface modification.

Tumor targetability is one of the most promising strategies for enhancing the PA signal in cancer imaging. The tumor microenvironment (TME) is characterized by high H_2_O_2_ and glutathione (GSH) levels, low pH value, and hypoxia; therefore, the incorporation of TME-responsive NM suggests potential for tumor-specific PAI. Yan et al. introduced Ce6-modified CoMn-LDH nanosheets for an enhanced tumor microenvironment response [106]. The CoMn-LDH nanosheets exerted complete quenching of ^1^O_2_ generation by the browning of the particle. The results indicated a good linear correlation between PA signal intensity and Ce6/CoMn-LDH concentration within 0–100 μg/mL. In vivo injection of the Ce6/CoMn-LDH nanosheets produced a weak signal that was observed after 2 h injection and increased gradually from 2 to 12 h after injection, indicating the accumulation of Ce6/CoMn-LDH nanosheets in the tumor area. The results demonstrated that Ce6/CoMn-LDH nanosheets accumulated in the tumor owing to the enhanced permeability and retention (EPR) effect, which presented as PA-combined multimodal imaging agents.

rGO, a derivative of graphene, has also been adopted as a promising PA probe thanks to its high drug loading capacity and optical absorption property in the NIR regime. Chen et al. fabricated an indocyanine green (ICG)-conjugated rGO nanocomposite for dual use of PAI and fluorescence imaging (Figure 7C) [107]. The nanocomposite was shown to exhibit little toxicity and high PA/fluorescence signals both in vitro and in vivo (Figure 7D). ICG-rGO nanocomposites showed high cytocompatibility at a concentration of 100 µg/mL and there was no obvious tissue damage in the heart, liver, spleen, kidney, or lung after injection of the particle. Moreover, ICG-rGO nanocomposites induced 63.5% of cellular uptakes and then exhibited strong red fluorescence. In vivo fluorescence imaging and PA imaging also demonstrated that ICG-rGO nanocomposites accumulated in the tumor and significantly enhanced the imaging contrasts. rGO is considered a promising candidate as a multimodal imaging probe for cancer diagnosis.

### 4.2. Photoacoustic Imaging for Monitoring Phototherapy

**Figure 8:**
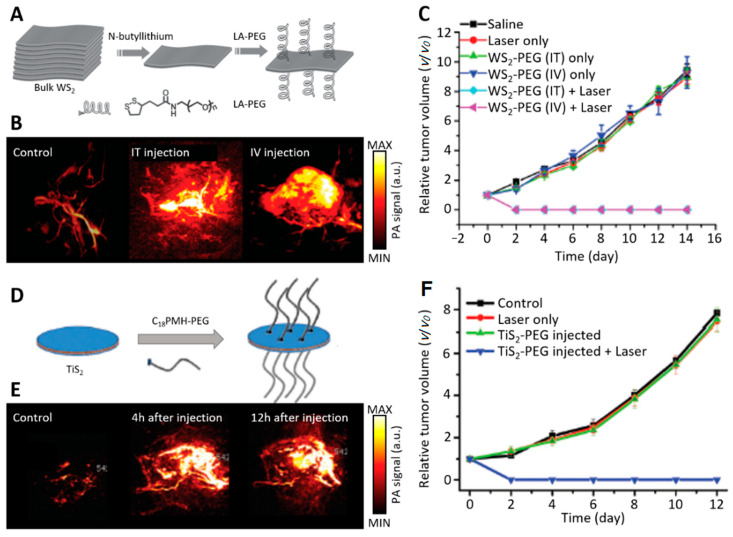
(**A**) Schematic diagram of exfoliation and PEGylation of WS_2_ nanosheets. (**B**) In vivo PA images of 4T1 tumor bearing Balb/c mice before, after IT injection, and after IV injection of WS_2_ nanosheets. (**C**) The growth of 4T1 tumors in different groups of mice after various treatments indicated. The relative tumor volumes were normalized to their initial sizes. (**D**) Schematic diagram of TiS_2_-PEG nanosheets. (**E**) In vivo PA images of 4T1 tumor bearing Balb/c mice before, 4 h after, and 12 h after IV injection of TiS_2_-PEG solution. (**F**) The growth of 4T1 tumors in different groups of mice after various treatments indicated. The relative tumor volumes were normalized to their initial sizes. PA, photoacoustic; IT, intratumoral; IV, intravenous. The images are reproduced with permission from [76,128].

As an emerging phototheranostic modality, interest in PA-combined PTT has increased in recent tumor treatment research [76,128,129,130]. After PAI provides guidance towards the tumor site and reveals strong tumor contrast after injection, the targetability and efficiency of PTT can be significantly increased (Figure 8). Several 2D nanomaterials have multimodal characteristics with PT and PA properties; therefore, they have been extensively studied for PAI-guided PTT. AM, the 2D layered material exfoliated from bulk antimony (Sb), has been highlighted because of its superior physicochemical properties compared with typical 2D nanomaterials such as graphene, MoS_2_, WS_2_, and BP [131]. Yu et al. fabricated liquid phase exfoliated AM nanoflakes (AMNFs) exhibiting unique PA properties, making AMNFs superior to both other 2D materials, such as BP, GO, and TMDs, and commonly used PA contrast agents, such as indocyanine green (ICG) and gold nanorods (AuNRs) [108]. The AMNFs exhibited 42.36% PT conversion efficiency, which was about two times higher than other 2D NMs (e.g., GO 25%, BP 28%, MoS_2_ 28%, AuNRs 21%). The PA performance of 1000 µg/mL AMNFs exhibited a limit of detection (LOT) of 7.4 × 10^4^ cells, which correlates with a 0.74 mm^3^ tumor. It is known that about 85% of the human tumor is composed of 10^8^ cells of 1 cm^3^ [132]. Therefore, it can be demonstrated that the PA performance of the AMNFs is very sensitive. Sb_2_Se_3_, a chalcogenide formed from antimony and selenium, has also emerged as a promising PA-PT multimodal agent thanks to its attractive properties such as a proper optical bandgap (1.1–1.3 eV), high light absorption coefficient, low toxicity, and abundant material reserves [133]. However, Sb_2_Se_3_ has a unique crystal structure that inherently tends to grow into a one-dimensional structure, which makes it difficult to fabricate Sb_2_Se_3_ with the desirable 2D planar nanostructure. Zhou et al. reported on the construction of Sb_2_Se_3_ nanosheet highly efficient PA-guided PTT agents by NIR laser activation on mice with 4T1 tumor cells were inoculated on their thigh (Figure 8) [109]. The Sb_2_Se_3_ nanosheets were fabricated using liquid nitrogen pretreatment and a freeze–thawing approach, resulting in a high PT-conversion capability agent (extinction coefficient: 33.2 L/g·cm; photothermal conversion efficiency: 30.78%). Moreover, the poly(vinyl pyrrolidone) (PVP) surface engineering enhanced the biocompatibility and physiological stability, which led to a prolonged and 7.3 times increased PA signal after 6 h of in vivo injection compared with that of the initial value of the tumor. AM-based 2D NMs are promising PT-PA agents, possessing great PA properties and PT-conversion efficiency.

The direct exfoliation of 2D ultrathin ceramic nanosheets from layer-structured solid and hard MAX-phase ceramics has been introduced, which has been termed MXene by Gogosti et al. [134]. The ultrathin planar structure of MXene endows the sheets with a large surface area, providing not only abundant anchoring sites for therapeutic drugs, but also high PT and PA conversion efficiency. Han et al. explored the PA and PT performances of Ti_3_C_2_ Mxene [94]. These Ti_3_C_2_ MXenes exhibited a PA signal at an excitation wavelength of 808 in vitro and a significant increase in PA signal at a concentration of 15 mg/kg (particles per 4T1-beared tumor), indicating the potential for PAI-guided therapy. Ti_3_C_2_ Mxene also showed high in vivo compatibility and easy excretion out of the body, suggesting potential high biosafety for further clinical translation. However, MXenes are limited in respect to their use as diagnostic imaging agents because of their simple material composition. Several studies have been carried out to endow them with specific functionality for theranostic application. Dai et al. fabricated SP-modified MnOx/Ta_4_C_3_-based nanosheets [111]. The PT-conversion performance of the SP-MnOx/Ta_4_C_3_ composite nanosheets showed fast enhanced PA signals (highest optical density at 15 min) after subcutaneous administration in 4T1 tumor-bearing mice. Moreover, the SP-MnOx/Ta_4_C_3_ nanosheets exhibited contrast-enhanced properties in both CT and MR, suggesting the potential use of SP-MnOx/Ta_4_C_3_ nanosheets for multiple imaging tool-guided therapy.

As the other class of 2D-layered NMs, Bi_2_Se_3_ has been highlighted as a remarkable thermoelectric and photoelectric agent possessing suitable bioactivity and biocompatibility [135,136,137]. Xie et al. fabricated PVP-encapsulated Bi_2_Se_3_ nanosheets possessing an extinction coefficient of 11.5 L/g·cm at 808 nm, showing a PT conversion efficiency of 34.6% and excellent photoacoustic performance (about a twofold increase after 5 h of injection) [112]. The PVP-Bi_2_Se_3_ nanosheets showed high cytocompatibility in vitro (about 95% of cell viable after 48 h of 200 ppm PVP-Bi_2_Se_3_ treatment) and almost perfect in vivo clearance (in the liver, spleen, kidney, lung, and heart) after 30 days of intravenous injection.

MoS_2_, one of the novel 2D nanomaterials, exhibits unique visible photoluminescence with high absorption in the NIR range. Despite the superior optical properties, the use of MoS_2_ nanosheets for in vivo theragnosis agents is hampered by their instability and low intracellular delivery efficiency [138,139]. To overcome these issues, Shin et al. introduced HA-conjugated MoS_2_ nanosheets to induce HA receptor-mediated endocytosis of the nanosheets [115]. HA is one of the biopolymers used for cancer cell-specific targeting because overexpressed cancer cell receptors such as cluster determinant 44 (CD44), hyaluronan receptor for endocytosis (HARE), and lymphatic vessel endothelial hyaluronan receptor-1 (LYVE-1) are reactive to the HA [140,141]. Therefore, HA-MoS_2_ conjugates experience receptor-mediated endocytosis and intracellular disulfide cleavage by pH and GSH, and subsequently, HA degrades and MoS_2_ nanosheets accumulate in the cells. In this method, PAI in tumor-bearing mice showed a 1860-fold increase in PA signal compared with control groups, which resulted from the high light-to-heat conversion efficiency of HA-MoS_2_.

Pd nanosheets, as typical 2D nanomaterials with well-defined and controllable sizes, are promising candidates in PA-guided PTT owing to their strong NIR absorption and excellent biocompatibility [142,143,144]. Zhu et al. conjugated DOX, Pd, and ZIF-8 in a self-assembled one-pot method, and coated them with PDA to improve biocompatibility (DOX/Pd@ZIF-8@PDA) [118]. The prepared DOX/Pd@ZIF-8@PDA showed a ninefold increase in PA signal after 400 ppm NMs were injected in tumor-bearing mice. Moreover, as the concentration increased from 0 to 800 ppm, the temperature rose from 25 to 54 °C after 808 nm laser irradiation, indicating a high PT conversion efficiency for DOX/Pd@ZIF-8@PDA. Interestingly, the introduction of an 808 nm laser could significantly accelerate the release of DOX from DOX/Pd@ZIF-8@PDA. Hence, DOX/Pd@ZIF-8@PDA can be adopted as multimodal theranostic agents, which is feasible for PA-guided PTT and controlled drug delivery, making them ideal candidates for a smart and multifunctional theranostic platform.

## 5. Conclusions and Perspectives

PA imaging has emerged as a premier preclinical biomedical imaging technique with various potential configurations. Moreover, many studies have investigated the clinical translation of PA techniques. Although there are limitations in respect to clinical translation at this time, PA imaging may be advantageous for diagnosing diseases and monitoring therapeutic prognoses. As a potential clinical monitoring system, PA images have been used for monitoring phototherapy prognoses using the delivery of 2D nanomaterials that have strong optical absorption properties. To achieve a strong optical contrast in PA images, various structures of 2D nanomaterials have been investigated and showed high optical contrast in PA images as well as a promising therapeutic effects. For future clinical translation and commercialization, 2D nanomaterials should meet requirements such as low toxicity, effective clearance, and high biodegradability. In addition, PA imaging systems need to be improved to achieve a higher signal-to-noise ratio in deep tissue. Because a laser source with a tunable wavelength is costly, 2D nanomaterials that absorb the 1064 nm wavelength (the default wavelength of Nd:YAG laser) would be a good option for successful commercialization through reducing the system cost.

## Figures and Tables

**Table 1:** Performance benchmarks of representative PA imaging systems. PA, photoacoustic; OR-PAM, optical resolution photoacoustic microscopy; AR-PAM, acoustic resolution photoacoustic microscopy; PACT, photoacoustic computed tomography; FOV, field of view.

Type	Lateral Resolution	Axial Resolution	Imaging Depth	Imaging Time	FOV	Ref.
OR-PAM	2.56 μm	-	1.2 mm	70 min	7.8 × 10 mm^2^	[31]
	3.6 μm	27.7 μm	1 mm	7 s	9 × 4 mm^2^	[34]
	2.7 μm	46.4 μm	400 μm	4 min	6 × 8 mm^2^	[35]
AR-PAM	590 μm	150 μm	25 mm	20 min	60 × 32 mm^2^	[39]
	45 μm	33 μm	7.6 mm	10 min	9 × 7 mm^2^	[40]
	84 μm	38 μm	2.3 mm	224 s	36 × 80 mm^2^	[41]
PACT	250 μm	100 μm	13 mm	16 s	25 × 30 mm^2^	[42]
	1.5 mm	-	10 mm	0.1 s	20 × 20 mm^2^	[44]
	1.2 mm	205 μm	30 mm	0.2 s	40 × 60 mm^2^	[27]

**Table 3:** Recent photothermal (PT)-Photoacoustic Imaging(PAI) multimodal studies classified by their characteristics.

PT-Multimodality	Key Material	Formulation	Modification/Functionalization/Hybridization	Experimental	Ref.
x	Black phosphorus (BP)	Quantum dot	Sulfonic ester of the titanium ligand (TiL4)	MCF-7, 293T cells, and MCF-7 tumor-bearing Balb/c nude mice	[103]
	Layered double hydroxide (LDH)	Nanosheet	CoMn and chlorin e6 (Ce6)	HeLa, U87mg, HepG2, 4T1 cells, and HeLa tumor-bearing Balb/c nude mice	[106]
	Reduced graphene oxide (rGO)	Nanocomposite	Polyethylene glycol (PEG), indocyanine green (ICG)	Hela cells, Balb/c nude mice	[107]
o	Antimonene (AM)	Liquid phase nanoflake	PEG	293T, MCF-7, SK-BR3, T47D cells, and MCF-7 tumor-bearing mice	[108]
	Antimony (III) Selenide (Sb_2_Se_3_)	Nanosheet	Poly(vinyl pyrrolidone) (PVP)	4T1, MBA-MD-231 cells, Balb/c nude mice	[109]
	Tantalum carbide (Ti_3_C_2_ MXene)	Nanosheet	SP (soybean phospholipid) and doxorubicin (DOX)	4T1 cells, 4T1-inoculated mice	[94]
	Tantalum carbide (Ta_4_C_3_ Mxene)	Nanosheet	MnO, SP	4T1 cells, Kunming mouse, 4T1 tumor-bearing mice, and Balb/c nude mice	[110]
	MXene (Ta_4_C_3_)	Nanosheet	Manganese oxide nanoparticles (MnO_x_) and SP	4T1 cells and 4T1 tumor-bearing nude mice	[111]
	Bismuth selenide (Bi_2_Se_3_)	Nanosheet	None	MCF-7 cells and MCF-7 tumor-bearing Balb/c nude mice	[112]
	Bi_2_O_2_Se	Quantum dot	None	A549, MCF-7 cells, and MCF-7 tumor-bearing Balb/c nude mice	[113]
	Bi_2_Se_3_	Nanodish	HA, polypyrrole (PPy), and zinc phthalocyanine (ZnPc)	4T1 cells and 4T1 tumor-bearing Balb/c nude mice	[114]
	Molybdenum disulfide (MoS_2_)	Nanoconjugate	Hyaluronic acid (HA)	HCT116 cells and HCT116-inoculated mice	[115]
	MoS2	Nanosheet	Iron oxide nanoparticle (IONP) and PEG	Balb/c mice and 4T1 tumor-bearing mice	[116]
	MoS_2_ and Bi_2_S_3_	Nanosheet	PEG	L929 cells and 4T1 tumor-bearing Balb/c nude mice	[117]
	Palladium (Pd)	Nanosheet	DOX, Zeolitic imidazolate frameworks (ZIF-8), Polydopamine (PDA)	WBCs (white blood cells) from mice, 4T1 cells, and 4T1 tumor-bearing mice	[118]
	Pd	Nanoplate	Au and PEG	4T1 tumor-bearing Balb/c mice	[119]
	Titanium (Ti)	Nanosheet	PEG	SMMC-7721, B16, J774A.1 cells, and Balb/c nude mice	[120]
	Germanene	Quantum dot	PEG	MCF-7, 4T1, H1299, HeLa cells, and Balb/c mice	[121]
	Boron	Nanosheet	PEG	HeLa, PC3, MCF-7, A549 cells, and MCF-7 tumor-bearing mice	[104]
	Manganese boride	Nanosheet	Bi	4T1 cells and 4T1 tumor-bearing mice	[122]
	MnO_2_	Nanosheet	None	U87MG cells and U87MG tumor-bearing mice	[123]
	Tungsten disulfide (WS_2_)	Nanosheet	PVP	L929, HT29 cells, KM mice, and HT29 tumor-bearing KM mice	[124]
	Co_9_Se_8_	Nanoplate	Poly(acrylic acid) (PAA), DOX	HepG2 cells, HepG2 tumor-bearing Balb/c-nude mice	[125]
	Au	Nanoring	None	Raw 264.7 cells, U87MG tumor-bearing nude mice	[126]
	Cu_2_MnS_2_	Nanoplate	Monomethoxycarboxyl polyethylene glycol (mPEG-COOH)	HeLa, S180 cells, and S180 tumor-bearing Balb/c nude mice	[127]

## Data Availability

No new data were created or analyzed in this study. Data sharing is not applicable to this article.
